# Supplementary material for: Genome-Wide Identification and Expression Analysis of CAMTA Genes in Cassava Under Abiotic Stresses
Source: Plants (Basel). 2025 Dec 8;14(24):3743. doi: 10.3390/plants14243743 (PMC12736797; doi:10.3390/plants14243743)
Supplement: Supplementary file 1 [file plants-14-03743-s001.zip › Appendix .pdf]

plants

MDPI

Appendix A

Table S1. qPCR Primer List

Gene name	Sense primer (5'-3')
MeCAMTA1-F	GTTGGCTTGTAGTGAAGTGCGA
MeCAMTA1-R	CACAGACAATAACCTCTCAAGTCGTAG
MeCAMTA2-F	CTGGGCATCATATTTTGGGAG
MeCAMTA2-R	GAACCCAGCTATTCCTTTATGTCC
MeCAMTA3-F	CACGACATAGATGGCTACGACC
MeCAMTA3-R	TGAGGTTCAGGAGCAACACG
MeCAMTA4.1-F	TTCCATAGAAGTCAGTTCGGAGAT
MeCAMTA4.1-R	TCCTCCAGTCTTCTCAAATATTGACTA
MeCAMTA4.2-F	TCCTGTAAAGTGTCATCTGGTGTG
MeCAMTA4.2-R	TGAGTTCCCAAGACAGGAGAGTATA
MeCAMTA6-F	CGCAGGTGTTATTGGCTACTTGAT
MeCAMTA6-R	ACCTCCTGAGTTTCCCGATAGTG
MeACTIN-F	TGGATTCTGGTGATGGTGTGAGT
MeACTIN-R	CCGTTCAGCAGTGGTGGTGA

Table S2. Ka, Ks and Ka/Ks of *MeCAMTA* gene family.

Seq1	Seq2	Ka	Ks	Ka/Ks
MeCAMTA4.1	*MeCAMTA4.2*	0.1001	0.3647	0.27

Appendix B

Figure S1. Cassava of the 10 conserved motifs of MeCAMTA proteins.

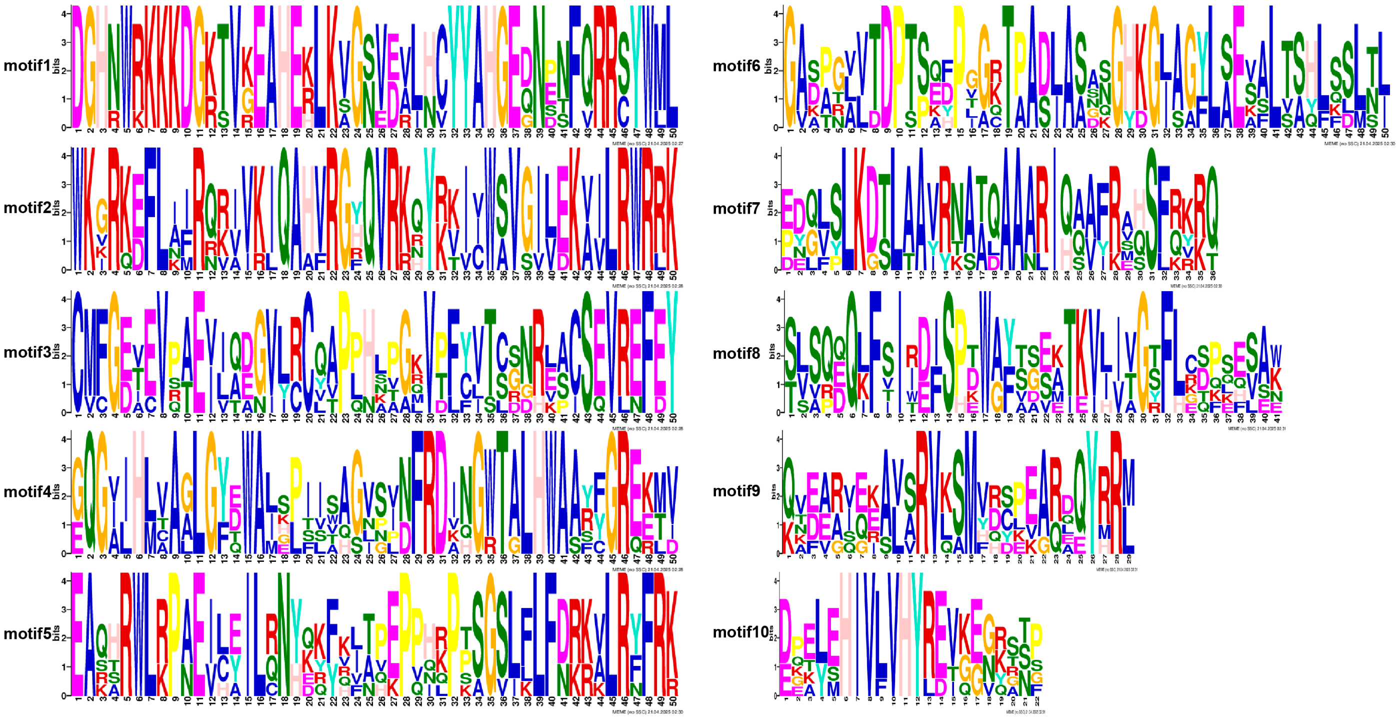

Figure S2. Collinearity analysis between cassava and solanum tuberosum.

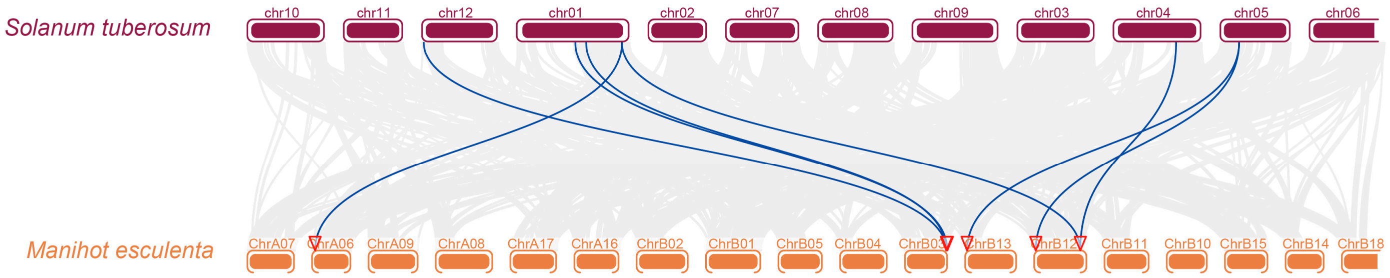

Figure S3. Cassava drought transcriptome analysis.

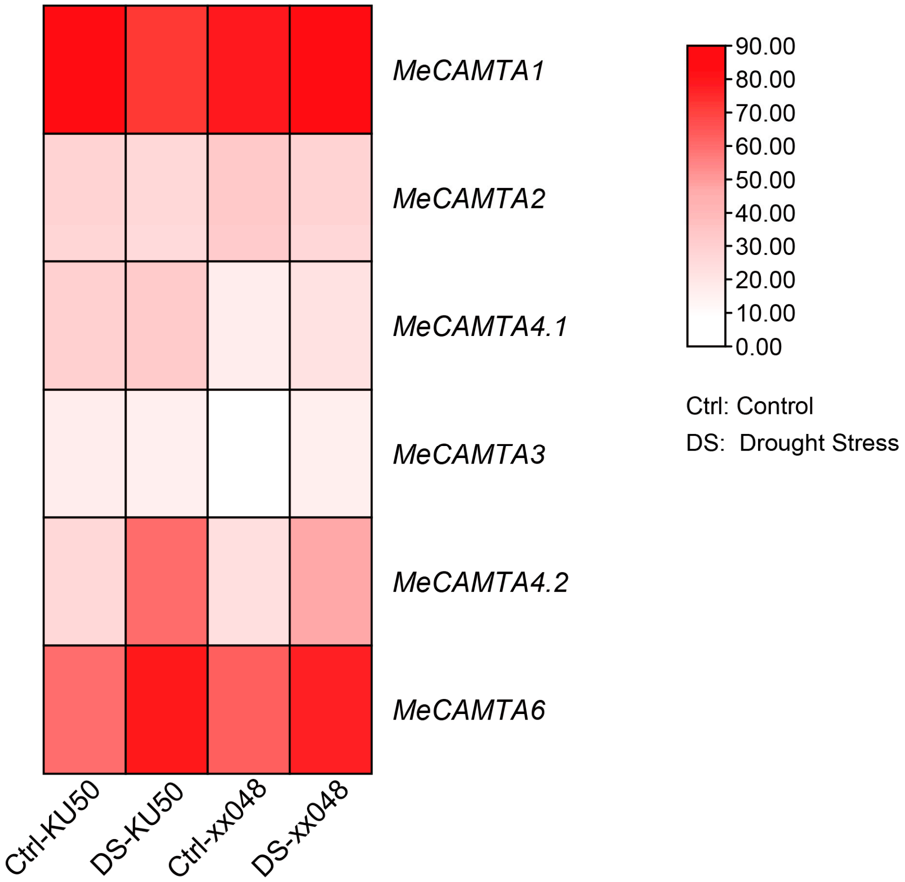

The original CDS sequences of the six MeCAMTA gene family members

>MeCAMTA1 (DescChrA06G00699830.1) +

ATGGCTGGTCGCGGATCGTATGGACTGGGCCCTCGATTAGATATTCAACAATTATTTATA
GAAGCACAACACCGATGGTTGAGACCTGCTGAGATTTGTGAAATTCTTCGTAATTATCAA
AAGTTCCATATTGCTCCAGAGCCTCCAAACAGGCCACCGAGTGGTTCTCTCTTTCTTTTT
GATCGGAAGGTTTTGAGGTACTTTAGAAAAGATGGACATAATTGGAGGAAAAAAAAGGAT
GGAAAGACCGTGAAGGAAGCTCATGAGAAGCTGAAGGTTGGAAGTGTTGATGTATTGCAC
TGCTATTATGCCCATGGAGAAGATAATGAGAACTTTCAAAGGCGCAGCTATTGGATGCTT
GAACAGGAACTGATGCACATAGTTTTTGTCCACTACTTGGAAGTGAAGGGTAACAGGACA
AATAGCAGCCCTTCAAATAGCTTAGCTGCAAGTTATAACAAAGAGCCTTCAGGAAATACA
GATTCAACAAGCCCAACTAGCACTCTTGCATCCTTTTGTGAGGATGCTGATTCAGCGGAT
AGTCAGCAATCAAGTGCTGGACGCCACACTTTTCTTGAGTCACCACAAATGCAGAGTAAT
CCTGCAATAGACAAAATAAATGCTGGTGTTTTGAGTTCAGAATTTCTGCATCATGGCTCA
GATAATCGAGAAGTTCGGTCATCAAATCCTGTATCAGGTTCAGTCTCCTATGTTCACAGA
GATGGACCTGGAGCTAATGGTGGTACTTGCATTACTGAATCTGAGAGCTTGGCCTCCTGG
GAGGAGGTCTTGGAACAATATGCAGTGGGAAACAAAAATGCAGCTTCCAATTTATCAGTG
ACTTCCAATCACTCTAATCCTACTGGTATTGGAAGGAATGAGATTTTCAGTGAGATTTTA
GCTGGTGGGGGTGCTGCTAAAGATGAGCTCAGTGGTTCTTTGACAATGGAATCGCATTGG
CAGATTCCTTTCGAGAACAGTTCCTTGCACTTGCCAGAGGGCTCTCTTGACCAGACACTA
GATTTGGAATTTGCGTACAATTTGGATCCTAGGTTTTTTGATCAAAGAGCTCATAATGTG
GATCTTCAAAATGCATTTGATGAAATTTTCTCTTGTGCTGTTCAGCATAATGAAGAACTT
GTGCAGAACAACCTTCAAATGCAGCTTGCAAATTCAGAACCACATCTCAGTATGCAGACA

AAATCTGAGAATGAAATTTCTGTGGGAGAAAATAATATATATGCTTTTAAACCAGCATTA
TTAGGTGGAGAAGAGGGCTTGAAAAAAGTTGACAGCTTTTCTCGGTGGGTTACTAAAGAA
CTTGGAGAGGTGGATGATTTGCATATGCGGTCCTCATCTGGTCTTTCATGGAGCACTGTT
GAATGTGGAAATGTGGTAACTGAAGAATCATTGAGCCCCTCTCTTTCACAAGACCAACTT
TTCAGCATAATTGATTTTTCACCAAAGTGGGCATATGCAGACTCAAAAACAGAGGTTCAT
ATTACTGGAACATTTTTGAGGAGTCAGCAAGAGGTGGCAAAATATAACTGGTCTTGCATG
TTTGGGGAAGTTGAAGTACCTGCTGAGGTTTTGGCAGATGGGATTCTTTGCTGCTATGCT
CCGCCTCACAATGTTGCAAGAGTTCCTTTTTATGTTACATGTTCCAACAGGTTGGCTTGT
AGTGAAGTGCGAGAATTTGATTATCAGGTGGGCTCTGCTCAAGATGTAGATGTCAAAGAA
GTTTATAGCGCTAGTATCAATGATATGCATCTTCATCTACGACTTGAGAGGTTATTGTCT
GTGAGATCCTCTAGTGCTCCAGATTGTCTCTCTGACGGTGCTAGAGCGAAGCAGAGGTTG
GTCAGTGAGATAATTTTATTGAGGGAGGAGGATGAAGGATGCCCAATTGCAGAACCAACC
CCTGAGAGACCTCTATCCCAAGATGAAATAAAATGGCAGGTCCTTCAGAAGGCAATGCAA
GAGAAGTTGTATTCATGGCTTCTTCATATGGTAGCCGAAAATGACAAAGGGCCAAGTGTC
TTGGATGATGAGGGGCAAGGTGCACTACATTTAGTAGCTGCTCTTGGTTATGACTGGGCT
ATAAAACCAAGCATAACTGCTGGAGTCCCTATCAATTTCCGTGATGTGAATGGATGGACA
GCTCTCCATTGGGCTGCATTTTATGGCAGGCAAGAACTAGATACAGTTGCTGCCCTTGTC
TTGTTGGGTGCAGATACTCGCGTATTGACAGATCCATCTCCAGAATTTCCTTTGGGTAGA
ACTCCGGCAGACCTTGCTTCAGGCAATGGACACAAAGGAATCTCTGGCTTCCTTGCAGAG
TCAGCTTTGACGAGCTACCTACAATTGCTCACTCTAAATGATCCAAAGGAAGGTGTTGCG
CCAGATGTCTCAGGAATGACTGCTGTGCAAACAATTGCAGAGCGGATGGCTACCCCTGTG
AATGAAACTGATGTGCCTAATTTGCTACCACTGAAGGATTCCCTGACTGCTATTCGGAAT
GCCACGCAAGCAGCTAATCGTATACATCAAGTATTCAGGATGCAGTCATTCCAGCGGAAA
CAGTTAACTGAGAGTGGTGGTGATGGATCCAATATATTTGATGAGCGAGCTCTTGCACTT
ATAGCTGGCAAGACAAACAAGCCTTCTGATGGACTAGCCAATGCTGCAGCTATCCAGATC
CAGAAGAAGTTCCGTGGTTGGAAGAAGAGAAAAGAATTCCTGATAATTCGGCAAAGAATT
GTTAAATTACAGGCCCATGTAAGGGGACACCAGGTAAGGAAGCAATATAGAACAATCATC
TGGTCAGTTGGAATTTTGGAGAAGGTTATCTTGCGTTGGAGACGTAAAGGAAGTGGTCTG
CGGGGATTCCGTCGAGATGCACTTACTAAGGATTCTAATGTGCAATGTGTGCCTGCGCCT
GCACCTCCGCCTCCGCCTCCAAAGGAGGACGAATATGATTTCTGAAGGAAGGAAGGAAA
CAAAATGAGGAGAGGCAGCAAAAAGCACTATCCAGGGTGAAATCCATGTATCATTGTGAG
GAAGGACAAGCTCAATACAGGAGGCTGCTGACTTACTTTGAACAACTCCGGGAAACTACG
GAATCTGAAATGGTAGTGAGCAGTCCTAGTGAAATGAGGTATGGTGATGAAGAACTTTTT
GATTCACTTTTAGACGACGACACTTTCATGTCAATAGCATTTGAGTGA
>MeCAMTA2 (DescChrB03G00619160.1) -
ATGGCTGAGACTAGAAGATACTTTCCCAATCAACCACTTGACTTGGAGCAGATATTGGAG
GAAGCAAAACACCGTTGGCTTCGTCCGAATGAAATCTTGGAAATACTTCGTAACTATCAG
AGGTTTAAATTGACTCCAGAGCCACCCATTCAGCCTTCAGCTGGTTCTTTGTTTCTATTT
GATCGAAAAGCACTTCGATATTTTCGTAAAGATGGTCATCGATGGAGGAAGAAAAAGGAT
GGAAAAACTGTTAGAGAAGCTCATGAAAAGTTGAAGGCTGGCAGTGTGGATGTTCTTCAT
TGTTATTATGCCCATGGGGAGGACAATGACAGCTTTCAGCGGAGATGTTATTGGATGCTT
GACGGGAAATTAGAGCATATTGTCCTTGTGCACTACAGAGAAGTAAAGGAGGGATACAGG
TCTGGTGTATCTCATTTGCTAGCTGATCGAGGTACCCAGGTTGAAAGCCCTCAACCCATT
TCTGCTCCTTCCATTGCACAAACATCCTCACCTGCTTTTACAGCTCAAATGTCTTTTGCA

TCAAATCCAAATGAAATTGACCGGAATGGCCAAACATTGTCTTCAGAGTTTGAGGATGTT
GATTCCAGAGATAATGTAGGAGCTTGTCTCACAGAACAAAAGATTGGTTCTGTGTCACAT
AATGCTTCTTTACTTGCAGCTGAAGTTGAAGGATTCACTATGTTATCAACAAATGGAGTG
AAATTTGATCACAGCACTGAATCATCTTTGTGGGCTGAAATTCCTGGCTCCAGCAAGAAT
GCATATCATGTTCATGACCAGAAATTTTACGTTGGACAACCTAGAGGAGCTGACGTTATT
ACTCATAAATTGACATATTCTAGAATAGACAGTGATGTTCCAGATTCTGTTGCTACTGGA
GACAGATTGATCAATGATGTAGATGACCAAGCACAAGCAGCAATTCCTCAGAGATCAATT
CAGGAACATGATTTTAAGTTGGTTCCTAATTCCCAGTTCCATGATCATTCTGGCTCTCAA
ACAGCTGCTTCTATTGCACAAGTGGATAACAAACCCAAAGATGGTGGTGCAAGTACTAAT
GAATTAGGAGAGCTAAAAAAACTTGACAGTTTTGGAAGATGGATGGACAAAGAAATTGGT
GGAGATTGTGATGATTCTTTGATGGCCTCGGACTCTGGCAATTATTGGAATACGTTGGGT
CCTGAGAATGAAGATAAGGAAGTATCTAGTTTGTCACACCATATGCAGTTAGATATTGAA
TCATTGGGTCCTTCTCTTTCCCAGGAACAGTTATTTAGCATTCGAGATTTCTCTCCAGAC
TGGGCTTATTCTGGTGTTGAAACGAAGGTTCTAATCGTTGGTACATTTTTAGGGAGCAAG
AAGTTTTCTAGTGAAACAAAATGGGGCTGTATGTTTGGTGAAATTGAAGTTTCTGCTGAA
GTTCTCACAGATAATGTTATCAGATGTCAGGCCCCTCTTCATGCTACTGGGCAGGTCCCA
TTCTATGTTACCTGTCGTAACAGATTAGCTTGCAGTGAGGTGAGGGAATTCGAATATCGT
GAAAATGCATCAAGAGTTGCATCCATATCTAACAGCAGTTTGCAAGAGGAAGAACAGCGC
TTCCTAGTGCGTCTAGCAAAGTTGCTGCATTTGGGGCTGGAGAAGAAATGGCTGAATTGC
TCCATTGAAAGATGCAGCAAATGTAAAATAAGAAGTACCTTATATTCAATGAGAAATAAT
ATCGATAATGAATTGGCAAGGGCCAAGGAGAGTTGGATGGTCTCTGAAGTTAATTTCACA
GATGCTAGGGATAAATTTATCCAAAGTTTGCTAAGTGACAAGCTTTTTGAGTGGTTGGTC
TGTAAAGTTCATGGAGAGGGTAAAGGACCAGATATGTTGGATGGTGAAGGCCAAGGAGTT
ATACACTTGACTGCTGGTCTTGGTTATCAATGGGCCATGGGCCTTATAGTTGCTGCCAGT
AACAATCCCAATTTCAGGGATGCACAAGGAAGAACAGGACTCCACTGGGCATCATATTTT
GGGAGAGAAGAAACAGTCATCGAACTGGTTAGATTGGGTGTGGATCCAACTCTTGTTGAT
GATCCAACCTCAGCATTTCCTGGAGGACAAACTGCGGCTGATTTAGCATCAAGCCAAGGA
CATAAAGGAATAGCTGGGTTCTTGGCTGAAGCATTTTTAACTAGTCACCTTTCTTCATTA
AATATCAAAGAAAATATAACAGATACTATTGATGCAACCATTGCAGCAGAAAAGCCAACT
GAAGCTGCAGCCCAAGTTGCCTTTCCATTGGATGGCGGAGCAGATGATGGGTTTTCCTTG
AAAGGAACTCTTGCTGCTGTCAGAAAATCAACTCTTGCAGCAGCTCTTATTCAAGCTGCC
TACCGGTCTTCTTCATTCCGATATAGACAATTTCCTAAGAGCAATGATGATTCTGAAGTT
TCACTTGACCTAGCTGCTCTTGGTCCTTTGAACAAATATCAACGGAGGAGTGATTTTGAA
GATTATTTGCATTCTGCAGCTGCAAGGATCCAACAAAAGTATCGTGGCTGGAAGGGAAGA
AAAGAATTTTTGAAGATACGTAATCGAATTGTAAAAATTCAGGCTCATGTGAGGGGACGT
CAAGTACGCAGGCAGTATAAAAAGGTTATTTGGTCTGTTAGTATTGTTGAAAAAGCAATA
CTGCGATGGAGGCGAAAAAGATCTGGCTTGCGAGGATTTCGGTTGGAAAAGCTATGTGGA
GATGTGATTCAGGGGACTGAAAAAACTGATGAGTATGAGTTTCTGAGGATTGGCCGCAAA
CAGAAATTTGCTGGAGTTGAAAAAGCGTTGGCAAGAGTCAAGTCCATGGTCCGAGACCCT
GTAGCACGTGACCAGTACATGAGGCTAGTTACAAAATCTGAGAATCTCAAGATGAATAAT
GGAGAGATTAATGTATCACCGCAAGATTTAAGTTGA
> MeCAMTA4.1 (DescChrB12G00091490.1) +
ATGTCGCAATCAGGGTATGATATCAATGTTCTGTTTCAAGAAGCTCAAGCACGATGGTTG
AAACCTGCGGAAGTGCTCTATATATTGCAAAACCATGACAAGTACCAGCTCACTCAGGAG

CCACCTCAAAAGCCAACTAGTGGGTCTTTATTTCTTTTCAATAAAAGGGTCCTTCGTTTC
TTTCGCAAAGATGGTCATAATTGGCGTAAAAAAAAGGATGGAAGAACTGTTGGGGAAGCA
CATGAACGACTCAAGGTTGGAAATGTTGAGGCTTTAAATTGTTATTATGCACATGGAGAG
CAAAACTCAAATTTTCAGAGACGTAGCTACTGGATGCTGGACCCGGCGTACGAGCATATT
GTTCTTGTTCATTATAGAGAGATTGGTGAGGGAAAGTCCACTCCTGGACCTGCTGCACAG
TTATCACCAGGGTTTTCTTCCTCTTTCAGCCCAAGTCAAACATCTTATACTACTCAGAAT
CCAGATTCTACCTCTGCAATTACTGATAAATATGACCCATACCGGAATTCATCTAGTCCA
AGTTCCATAGAAGTCAGTTCGGAGATGGTTACCAAGGAAAATGGATTAGACGCTACAACA
GAATTTACTAGTTATAGAAAGGATGAGGTTAGTCAATATTTGAGAAGACTAGAGGAGCAG
TTGAGTCTGAACGAGGACAGCATCAAAGAAATTGATCCATTATGCAGTGAGGAAAGAGCC
ACAGATAATACAGAACTTCTGGAATATGAAAAGCAAATCCCCAAGGAGGATAACTCTGCA
AATTTGTTATTTAGACCAGAATATTTTGTGAATAATCAATCTTATGGTGGACATGCTGGA
ATGCAGCTGCAGACTAACAATCTTGTGCACCTTCAGGATGCAGGTGATAGTGGCAAATAT
GATCAATCGTATCTAGACAAGTATGCAGATGGGAATAATGAATCTGTGTCTTGGAATGAA
GTGTTGGACCCCAGTAAAGCTTCATCCGGTGCGGAGTACCAGGAAAAGCCCCAGCCTTCT
TTAAGGGGACCAGCTGAAGAGCATGAATATTCTGGTTGGCTAAATTTCAATGGAACTAAT
GCCAGAAATTCTTCCCTATTGCTGCATCAAGAAGTTGAAAATTTTGAAATTCCTGCTTAC
GCTCCTGTTATTGGAAGTCATGAAACCAATCCAGACTACTATTCAATGCTATACGACCCA
GGCCAGCTTGGAGTGCCAATTGAAGCAGATTCAAGTTTGACTGTTGCACAACAGCAGAAA
TTTATTATCTGGGAAATATCCCCAGACTGGGGTTTTACCTCTGAGGCTACAAAGGTAATT
GTTGTTGGATCTTTTCTATGTGACCCATCACAATCAGCATGGACATGCATGTTCGGTGAT
ACCGAGGTTCCAACTGAAATCATTCAAGAAGGTGTTCTCCGCTGTCAAGCACCTCCCCAT
CTTCCAGGAAAGGTCACTTTCTGCATTACTTCTGGCAATCGGGAGTCTTGCAGTGAGGTC
AGAGAATTTGAGTATCGAGCTAAGAGTAGTTGTCCTCATTGCAGCTTATCCAAAACAGAA
GTTGCTAAGAGTCCAGAAGAGTTGTTACTTGTCAGATTTGTGCAGTTTCTTCTTTCTGAT
CCCTCTTTGCAGAAAGAAGACAGCATAGAAACAGGAATTCAGCTAATGAGAAAATTAAAA
ACTGGTGATGATTCATGGGGAAGTATCATTGAGGCTCTCTTAGTTGGTAATGGAACTTCA
ACAGGCACTGTTGATTGGCTTCTTCAACAGCTTCTAAAAGACAAATTGCAGCAGTGGCTT
TCTTCCAAGTTTCAAGAAAGACAAGATCAACCTAGCTGTACCTTGTCCAAGAAAGAGCAG
GGGATCATACACATGGTTGCGGGGTTGGGTTTTGAGTGGGCCTTGAGCCCAATTCTCAGT
CATGGAGTCAGTATAGACTTCCGTGACATTAATGGATGGACTGCTCTTCATTGGGCTGCT
CGTTTTGGAAGGGAAAAAATGGTAGCAGCGCTTATAGCTTTGGGTGCATCAGCTGGGGCT
GTCACAGATCCCACATCACAGGATCCAATAGGCAAAACTCCAGCATCTATTGCTGCTAAC
AGTGGTCACAAGGGTCTTGCAGGTTATCTGTCAGAGGTGGCACTCACGAGCCATCTTTCA
TCCCTTACAATAGAAGAAAGTGAGCTTTCTAAAGGATCTGCTGAGGTCGAAGCTGAGAGA
ACTGTAGATGCCATCTCGAAGGGGAGTTTTGCTGTCAGTGAAGATCAGGTTTCACTTAAA
GATACTTTGGCTGCTGTTAGAAATGCAGCTCAAGCTGCTGCACGTATACAATCTGCTTTC
AGAGCACATTCATTCAGAAAGCGGCAAGAGAGAGAAGCTGCTACCTCTGCTTATAGTATA
GATGAGTATGGTGTCAATTCCAGTGATATACAAGGACTTTCAGCCATGTCAAAACTGGCA
TTTCGCAATGCACGTGATTACAATTCAGCTGCTTTATCTATTCAGAAGAAATATAGAGGA
TGGAAAGGTCGCAAGGATTTTCTAGCATTTCGACAGAAAGTTGTGAAGATACAGGCGCAT
GTGAGAGGTTATCAAGTAAGGAAGCACTATAAGGTAATATGCTGGGCTGTTGGAATTCTG
GATAAGGTTGTGCTGAGGTGGAGACGTAAAGGAGCTGGTTTGCGAGGTTTCCGGAATGAG
GCAGAACCCAATGACAATGATGATGAAAGCGAAGATGAAGATATTCTCAAGGTATTCCGC

AAACAAAAAGTTGATGTTGCTATCGGTGAGTCTGTCTCGCGTGTGCTGTCAATGGTTGAT
TCTCCGGAGGCACGCCTGCAATATCATCGGATGCTTGAACGTTATCGCCAAGCTAAAGCT
GAACTAGGCGAAACGAGTGAAGCAGCAGCAACTTCTCTTGCTGACATGGAAAATGATGAT
ATGTACCATTTCCAGTAG
>MeCAMTA3 (DescChrB12G00106750.1) -
ATGGCAGAGGCTAGAAGCTCCCCCATTGGTAACCAGCTAGATATTCTACAAATTCTTGCT
GAAGCACGACATAGATGGCTACGACCTGCGGAAATTTGTGAAATTCTTCGCAATTATAAG
CAATTCCGTGTTGCTCCTGAACCTCAACATAGGCCACCGAGTGGTTCACTTTTGCTTTTT
GACCGCAAGGCGCTGAGATACTTCAGAAAAGATGGGCATAACTGGAGGAAGAAAAAAGAT
GGGAAGACAGTTAAAGAAGCTCATGAGAAGCTCAAGTCTGGAAGTGTTGATGTTTTGCAT
TGTTATTATGCCCATGGAGAAGGAAATGAAAATTTTCAAAGACGGAGTTACTGGATGCTT
GAAGAGGAACTTTCTCATATAGTACTTGTCCACTATCGAGATGTAAAGGGAAACAGGGCA
AATTTTAATAATGTTAAGGAGCATGAAGATACTATTCCATGTACCCAAGAGATTGAAGAC
ACTGTACCCCATTCTGAGATGGATACTTCTGTTTCTTCCAATTTTCATCATGATAATTAT
CAGGTGCCTGCTCAAACTGCAGATACAATAAGCATGAATAGTGCGCAGGCGTCAGAATAT
GAGGATGCTGAATCAGTATATAATCACCAAGCAAGTTCTGGATTGCACCTTTTTCTTGAG
GAACAGCAACCTGCAAGAGAGAAGATAGATGCTAGCCTGCGTGACCGTCATGATCCTGTG
TCATTGTCAAGTGGTTATGAAGGGAAGTTGTCAGCAGTTCCTGGGAGGGACTTTTTCTCA
CTAGCCCATGCTGATAAAGCTGAAGACACTGAGGGTGCTAGCTCAACATTTCAGCTGCAG
AAACATTTCGACCTACCCTGTTGGAAGGACATATTAGAAAATTTTACTCCTGGAATTGAA
TCTGCATCCTTCCAGCCCCAATTTTCATCACAAGGCGATACTGCTGGAATCATTCCTAAA
CAAGAAGATGCAATACCGAAACAGCTTCTTTCCAACAGTTTACCGCTCCAGAAAGAGTGG
CAGAATTTGGAAGACGCTTCTTCACACCTCTCAAAGTGGGCCATGGATCAGAAGTTGCAT
CCAAATTCAACAGCTGATCTCACTACTAGTTTTCATGAACAAGAACTTCTCAGTGGTGAT
TTAACTAATACACTTGAGCTTTTTGGAACACAGAAAAGTGGTCATTCTGTGCAAAATGAT
GTTCAGTTACAATATTCCAATACAGACCAAAGTATAACCCCAGAAGGAAAATCCATATAC
TCTTCTACTGTGAAGCAGCTATCTGAAGAAGGTCTAAAGAAGCTTGATAGTTTCAGCCGA
TGGATGAGTAGGGAACTTGGTGATGTAAATGAGTCCCATATGCAGTCCAGTTCTGGGCCC
TACTGGGATGCAGTTGAAAATGAAAATGGGATTGACGATCCCAAAATCTCATCTCGAGTA
CACTTGGATACATATTTGCTAGGTCCTTCCCTCTCACAGGACCAACTCTTTAGCATCATT
GATTTTTCACCCACCTGGGCATATGTAGGCTCCAAGATTAAGGTTCTTATCGCGGGAAGA
TTCTTGAAGACTCCAGAAGAAGTAGAAAATTGTAAATGGTCATGTATGTTTGGGGAAGTT
GAAGTTCAAGCAGAGGTTATAGCTGATGGTGTTCTCCGTTGTCAGACTCCTTTAAATAAG
GCTGGCATGGTTCCTTTTTATGTTACATGTTCAGATAGAGTGGCATGTAGTGAAGTGCGT
GAATTTGAATACCGACTCAGTCAAGATGTGAATATTATTGATAGTTACAGCAGCAGCTCT
AGTGTAATGCGGTTTGGAAAATTATTGTCTCTTAACTCTCTTTCTCTTCCAAAATGCAAC
ACCAGCAATATAGTTGAAAATACTCAATTAAGCAATAAAATTAGTTCACTTCTGAAAGTG
CACAATGAAGAAGAATGGAATAAAATGTTAAAGCTTACTTCGGAAGCTGGAGTTTCCTTG
GAGAAAGTAAAGGAGGAACTTCTTCAAAAGCTACTCAAAGACAGGTTACATGTTTGGCTT
TTGCAAAAAGCAGCTGAAGGTGGAAAGGGCCCTAGTGTATTAGATGAAGGCGGTCAGGGA
GTACTACATTTGGCCGCTGCTCTGGGCTATGACTGGGCCCTTGAACCCACAATAGTTGCA
GGTGTAAGTGTCAATTTCCGGGACATAAATGGATGGACTGCACTTCACTGGGCAGCATCT
TGTGGCAGAGAGCGCACAGTTGCGTCCCTTGTCTTTCTTGGTGCAGCTCCTGGAGCATTA
ACAGATCCAACTCCTAAATATCCTACAGCCAGAACACCTGCTGACCTAGCTTCTGCTAAT

GGACACAAAGGAATTGCTGGTTATCTGGCAGAATCTGCTTTGAGCGCCCACCTTTTTTCT
CTTAATCTGGATAAGCAGGATGGTGATGTGGCAGGAGTTCCTGAGGCAAAAGCAGGGCAG
ATAGTTCCAGAGCATAGCACATCTGTAAATAGTGATGGAGACTTACCATATGGACTTTCC
TTAAAGGACTCATTAGCTGCTGTCTATAATGCTACTCAAGCTGCTGCTCGTATCCACCAA
GTCTTCAGGGTACAATCCTTCCAAAAGAAGCAGTTAAAAGAGTTTGGTGATGATAAATAT
GGAATGCCACATGAGCATGCTCTTTCATTTATTGCGTCTAAGGCACTCAAGTCTGGACAA
CATGATGAGCCCGTGCATGCTGCTGCAACAAGGATTCAAAACAAGTTCCGCAGTTGGAAG
GGTAGAAAGGAATTTTTAATAATCCGGCAGCGAATTGTTAAAATTCAGGCTCATGTAAGA
GGCCACCAGGTTAGGAAAAACTATAGAAAGATAGTATGGTCTGTGGGGATTGTGGAGAAA
GTTATCTTGCGTTGGAGACGAAAAGGAAGTGGTTTGCGTGGATTCAAATCAGAAGCACTT
ATTGGGGGTCCCAGTATGCAGGATAGATCACCAAAGGAGGATGACTATGATTTCTTTAAA
GAAGGCAGAAAACAAACAGAAGCAAGATCACAGATTGCTCTTGCGAGGGTGAAATCCATG
CACCAGTATCCTGAGGCAAGAGATCAATATCGCCGGCTGCTAAATGTGGTTACTGAGATT
CAGGAAACTAAGCTAGTGGGTGATCAAATTAACAATTCTGAAGCAACAGCCGAGTTCGAC
GACTTGATTGATGTTGAAGCATTATTTGATGATGACACTTTCATGCCTGCAGCTTCCTGA
>MeCAMTA4.2 (DescChrB13G00136590.1) +
ATGTCACAGTCAGGGTATGATATTAATGTTCTGTTTCAAGAAGCTCAAACGCGATGGCTG
AAACCTGCTGAAGTGCTATATATATTGCAAAACCATGAAAAGTACAAGTTTACCCACGAG
CCACCTCATAAGCCAACTAGTGGATCATTGTTTCTTTTTAACAAGAGGGTCCTTCGGTTC
TTTCGCAGAGATGGTCATAATTGGCGTAAAAAGAAGGATGGAAGAAGTGTTGGGGAAGCA
CACGAGCGACTTAAGGTTGGAAATGTCGAGGCTTTAAATTGTTATTATGCACATGGAGAA
CAGAACCCGAATTTTCAGAGACGTAGCTACTGGATGCTGGATCCGGAATATGAACATATC
GTTCTTGTTCACTATAGAGAGGTTGGTGAGGGAAAGTCTACTCCCAGATCTGCTGTGCAG
TTATCACCAGGGTTGTCTTCTGCTTTCAGTCCAAGTACAACATCTCATACTACTCACAAT
CGAGATTCTACCTCTGCAGTTAGTGATTTATATGACCCTGATCGGAGTTCATCTAGTCCA
AGTTCTACGGAAATCAGTTCAGAGATAGTCACCAAGGACAATGGATTAGAGACTTTAACT
GGGTTTACTAGTTCCCCAAAGGATGGCGTTAGTCAATTTTTGAGAAGGCTGGAGGAGCAT
TTGAGTCTGAATGAGGACAGCATTAAAGAAACTGACCCATTATGCAGTGAGGAAGGAATC
ACAAATGATCCAGAACTTCTGGAATTTGCAAAACAGATCTCCGAGAAGGATCACTATGTA
AATATGCTGCATGGACCTGAGAATATTGTGAATAATCAATGTTATGATTTTGGTGAACCT
CCTGGGTTGCAACTGCAGAGCAACAATGTTGTTCACCTACAGGACACAGGTGATGGTGGC
AAATACCATCAACCATTTGTAGAGTATGCAGATGGGAGCAAAGAATCCATTTCTTGGAAT
GAAGTGTTGGAGTCCTGTAAAGTGTCATCTGGTGTGGATTACCAGGAAAAGCCACAGCCT
TCTTTGAGGGAACCAGCTGAAGAGCATGAGTATTCTCATTGGCTAAACTTCAATGGAAAT
AATGTCAGAAATTCTTCTGAGTTGCTGCCTCAAGATGTTGAAAATTTTGATATTCCTTTA
TACTCTCCTGTCTTGGGAACTCATGAAACCAATCCTGACTACTATTCAATGCTATATGAT
GAAGGCCACCTTGGAGTGCCAATTGAACCTGATTCAAGTTTGACTGTTTCACGACAGCAG
AAATTTACAATCCGTGAAATATCCCCAGAATGGGGTTTTACCTCTGAGGCTACAAAGGTA
ATTATTGTTGGATCTTTTTTATGTGATCCATCAGAATCTGCATGGAAATGCATGTTTGGT
GAGACTGAGGTTCCCACTGAAATCATTCAAGAAGGTGTTCTCTGCTGTGTAGCTCCTCCC
CATCTTCCAGGAAAGGTCACTTTCTGTGTTACTTCTGGCAATCGGGAATCTTGCAGTGAG
GTCAGAGAATTTGAGTATCGAGCTAAGAGTAGCTGTCCTCATTGCAACTTAACCCAAATG
GAAGTTGCAAAGGGCCCAGAAGAGTTGCTACTGCTTGTCAGATTTGTGCAGATGCTTCTC
TCTGGTTCCTCTATGCAAAAAGAAGACAGCATAGAAACAGGAATTCAACTACTGAGAAAA

CTTAAAACAGATGATGGTTTGTGGAGTAGGATCATTGAGACTCTCTTAATTGGTAATGGA
ACTTCAACTGGCACCATCGATTGGCTTCTGGAACAACTTCTTAAAGACAAACTACAGCAG
TGGCTTTCTTTCAAGTCCCAGGAAAGACGAGATCAACCTAGTTGGACCTTGTCCAAGAAA
GAGCAAGGGATCATACACATGGTTGCTGGGTTGGGCTTTGAGTGGGCCTTGAGCCCAATT
ATCAGTCAGGGAATCGGTGTAAATTTCCGAGACATTAATGGATGGACTGCTCTTCATTGG
GCTGCTCGATTTGGAAGGGAAAAAATGATTGCAGCCCTTTTGGCCTTTGGTGCATCAGCT
GGGGTGGTCACAGATCCCACTTCACAAGATCCAGTTGGCAAAACTCCAGCATCCATTGCG
GCCGACAGTGGGCATAAGGGACTTGCAGGTTATCTTTCAGAGGTGGCACTAACAAGTCAT
CTTTCATCCCTTACACTAGGAGAGAGTGAACTTTCTAAAGGATCTGCTGAGGTCGAGGCT
GAGAAAACTGTAGATAGCATCTCAAAAGGGAGTTTTTCTGCCAATGAAGACCAGGTTTCA
CTTAAAGATACCTTGGCTGCAGTACGAAATGCAGCTCAAGCTGCTGCACGAATACAAGCT
GCCTTCAGAGCACATTCTTTCAGGAAGCGACAGAAAGAAGCTGCTATGTTGGCTAATAGC
ATAGATGAGTATGGCCTCAATTCCAGTGATATTCATGAGGTTTCAGCTATGTCGAAGCTG
GCCTTTGGCAATGCACATGACTACAAATCAGCTACTTTATATATTCAGAAGAAATATAGA
GGGTGGAAAGTTCGCCAGGATTTCCTAGCATTTCGACAGAAAGTTGTGAAGATACAGGCA
CATGTGAGAGGTTATCAAGTTAGGAAGCGGTACAAGGTAATATGTTGGGCTGTTGGAATT
CTAGAGAAGGCTGTGTTGCGGTGGAGACGCAAAGGAGTTGGTTTGCGAGGTTTCCGGAAT
GAGGGAGAAGCTATTGAAGATAGTGAAGATGAAGATATTCTCAAGTTGTTCCGCAAACAA
AAAGTTGATGCAGCTATTGAGGAGGCCGTATCACGCGTGCTGTCAATGGTTGATTGTCTT
GAGGCACGTCAACAATATCGTCGGATGCTTGAACGTTATCGCGAAGCCAAAGCTGAAGTA
ATTGAGACAAGTGAAGCAGCAGCAACTTCTGCCGACATGGAAAATGATGATATTTTACCA
TTTCAAGGAGGAGCAGCTTCTAACTATCTAACTTAA
>MeCAMTA6 (DescChrB03G00618330.1) +
ATGGAAAGTGGTGTGCAGGGGCTGCTTGTGGGTTCGGACATTCATGGATTTCACACGCTG
CAAGATTTGGATTATGGGAAAATAATGGAGGAAGCTAGTTCAAGATGGCTCCGACCAAAT
GAAATTCATGCAATACTTTGTAACTATAAGTATTTTGTCATTAATGTCAAGCCTGTACAC
TTGCCCAAAAGTGGTGTTATTAAGTTATTTGACCGTAAGAAGCTTAGGAACTTCCGAAAA
GATGGTCATAATTGGAAGAAGAAGAAAGATGGGAAGACTGTTAAAGAAGCTCATGAACAC
TTAAAAGTTGGTAATGAAGAAAGGATTCATGTATACTATGCACATGGGGAAGATAACCCA
ACCTTCGTCCGCAGGTGTTATTGGCTACTTGATAAGACTCTAGAACATATAGTCCTTGTG
CACTATCGGGAAACTCAGGAGGTGCAGGGTTCTCCAGTCACACCTGTGAATTCAAATTCT
AGTTCAGTCTCTGAACAGTCCCCTTGGCTTTTATCAGAAGAATTTGATTCTAGAGCTGGC
CATGCATATTATGTTGGTGTTAAAGAAGCTATAGATCCTGGTGACTGTTTAACTGTCAGA
AATCATGAAATGAGGCTTCATGAAATTAATACACTTGAATGGGATGAGCTTGTGACGAAT
GATCTTAACAATTCACACATGCCTAAAGAAGATAAGAATATGAGCTTTGACCAACACAAT
CAAATTGCAGTTAATGGGTCCAGGAATGATGGTATCCCTCTTCCAGTCTACAATTTATCT
GCAGAAGTTCCTCCTTTAGATTACTTAACTGAACCAATTGCAAGGAACAACACTTCTTGC
TTAAATATTCCAGAGGATGCTTACAGTAAGGCAACAAAAGTCCAAGGAAATTCAGATGTG
CAGGAAAAGGATGCTAGGATACTAGGGACTGGTGATTCTTTGGATGTGTTGGTTAATGAT
GGTCTACAAAGTCAAGATAGTTTTGGAAAGTGGATAAATGACATAATAGCTGACTCTGCA
GGTTCTGTAGATAATGCTTTGGTTGAATCTTCTATTTCATCTAGTCATGATTCTCACACT
TCTCCAGCAATCGATCAGTTGCAATCTTCTGTTCCTGAACAAATATTTGTCATCACTGAT
ATCTCCCACACTTGGGCCTTTTCAGCTGAAATGACAAAGATTTTAGTTACTGGATATTTC
CATGAACAGTTTCTACATCTAGCAAAATTGGATCTGTGTTGTGTGTGTGGAGATGCATGT

GTGCGTGCAGAAATTGTTCAGGCTGGGGTGTATCGCTGTTTGGTACCACAACATTCCCCT
GGATTAGTAGATCTGTTTTTGAGTTTGGATGGCCATAAACCCATAAGCCAAGTTCTAAAT
TTTGAGTACCGCCCTCCATTACGTGATCCTGTGGTTTCTTTAGAAGACAAACCCAAGTGG
GATGAGTTCAAACTTCAGATGAGACTTGCTTGCTTGCTTTTCTCTACATCCAGAAGCCTC
AGCATTTTAACCAGCAAGGTATCACCAGCTAACCTGAAGGAGGCTAAAAAGTTTGCCCAT
AAAACCTCTAGCATTTTCCATAGTTGGACATATTTGAACAAGTCAATTGAGGATGACAGA
GTGTCATTTTCACAAGCGAAAGATGGTTTCTTTGAACTCACTTTGCAGAACATGCTAAAG
GAATGGTTGTTGGAAAGAGTGGTTGAAGGCCGTAAAACCACTGAATATGATGCTCAAGGT
CAAGGAGCAATCCATTTATGCGCCCTTCTTGGATATACTTGGGCTATTCATTTATTTTCA
TGGGCAGGCTTGTCATTGGATTTCCGGGATAAACATGGATGGACAGCTCTTCATTGGGCA
GCATATTATGGAAGGGAGAAAATGGTTGCAGTTCTCTTATCTGCAGGGGCAAAGCCAAAC
TTGGTCACAGACCCCACATCAGAACATCCTGGTGGATGCACTGCTGCTGATCTTGCATCT
GCAAAGGGTTATGATGGCTTAGCAGCTTATCTGTCAGAAAAGTCTTTAGTAGCACAATTC
AAGGATATGTCTCTAGCCGGAAATGTTAGTGGCTCCCTGCAAACAAGTTCAACTGACAGT
ATAAATTCTGCGAACCTAAGTGAGGAGCAGCTTTATCTCAAGGATACTTTAGCAGCCTAC
CGAACAGCTGCTGATGCAGCAGCACGTATTCAGGCTGCAATTAAAGAACACTCCTTAAAA
GTTCGGACTAAAGCAGTTCAGGTTGCAAATCCAGAGGATGAAGCTCGAAATATAGTTGCA
GCAATGAAGATTCAACATGCCTTTCGCAACTATGAAACACGGAAAAGGATGGCAGCTGCT
GCCCGAATCCAGCACAGGTTTCGGACATGGAAAATCCGGAAGGAATTCCTTAATATGCGT
CGGGTTGCTATCAGAATTCAGGCTGCTTTTCGGGGCTTCCAAGTGAGAAAGCAATACCGC
AAGATAGTCTGGTCAGTTGGAGTGCTTGAGAAAGCAATTCTGCGTTGGCGTCTAAAAAGA
AAAGGCTTCCGTGGCCTTCATGTTGATCCTATTGAAGTAGTTGCGGACGAGAGGGAGGAA
AATGATGCAGAGGAAGACTTCTTCCAAGTTAGTAGGAAACAAGCTGAAGAGCGTGTTGAG
AGAGCAGTTGTACGGGTTCAAGCCATGTTCCGATCAAAGAAGGCACAAGAAGAATATCGA
AGGATGAAATTGACTTGTAACCAAGCAGAGCTGGAATATGGAGGGCTTCTTGACCATGAT
ATCGACTTGGACAGATGA

The original amino acid sequences of the six MeCAMTA members

>MeCAMTA1 (DescChrA06G00699830.1)
MAGRGSYGLGPRLDIQQLFIEAQHRWLRPAEICEILRNYQKFHIA-
PEPPNRPPSGSLFLFDRKVLRYFRKDGHNWRKKKDGKTVKEAHEKLKVGSVDVLHCYYAHGEDNENFQRRSYWM
LEQELMHIVFVHYLEVKGNRTNSSPSNSLAASYNKEPSGNTDSTSPTSTLASFCEDADSAD-
SQQSSAGRHTFLESPQMQSNPAIDKINAGVLSSEFLHHGSDNREVRSSNPVSGSVSYVHRDGPGANGGTCITESESLA
SWEEVLEQYAVGNKNAASNLSVTSNHSNPTGIGRNEIFSEILAGGGAAKDELSGSLT-
MESHWQIPFENSSLHLPEGSLDQTLDLEFAYNLDPRFFDQRAHNVDLQNAFDEIFSCAVQHNEELVQNNLQMQLA
NSEPHLSMQTKSENEISVGENNIYAFKPALLGGEEGLKKVDSFSRWVTKELGEVDDLHMRS-
SSGLSWSTVECGNVVTEESLSPSLSQDQLFSIIDFSPKWAYADSKTEVHITGTFLRSQQEVAKYNWSCMFGEVEVPAEV
LADGILCCYAPPHNVARVPFYVTCSNRLACSEVREFDYQVGSAQDVDVKEVYSASINDMHL-
HLRLERLLSVRSSSAPDCLSDGARAKQRLVSEIILLREEDEGCPIAEPTPERPLSQDEIKWQVLQKAMQEKLYSWLLH
MVAENDKGPSVLDDEGQGALHLVAALGYDWAIKPSITAGVPIN-
FRDVNGWTALHWAAFYGRQELDTVAALVLLGADTRVLTDPSPEFPLGRTPADLASGNGHKGISGFLAESALTSYLQ

LLTLNDPKEGVAPDVSGMTAVQTIAERMATPVNETDVPNLLPLKDSLTAIRNATQAANRI-
HQVFRMQSFQRKQLTESGGDGSNIFDERALALIAGKTNKPSDGLANAAAIQIQKKFRGWKKRKEFLIIRQRIVKLQA
HVRGHQVRKQYRTIIWSVGILEKVILRWRRKGSGLRGFRRDALTKDSNVQCVPAPAPPPPP-
KEDEYDFLKEGRKQNEERQQKALSRVKSMYHCEEGQAQYRRLLTYFEQLRETTESEMVVSSPSEMRYGDEELFDSLL
DDDTFMSIAFE*
>MeCAMTA2 (DescChrB03G00619160.1)
MAETRRYFPNQPLDLEQILEEAKHRWLRPNEILEILRNYQRFKLTPEPPIQP-
SAGSLFLFDRKALRYFRKDGHRWRKKKDGKTVREAHEKLKAGSVDVLHCYYAHGEDNDSFQRRCYWMLDGKLE
HIVLVHYREVKEGYRSGVSHLLADRGTQVESPQPISAPSIAQTSSPAFTAQ-
MSFASNPNEIDRNGQTLSSEFEDVDSRDNVGACLTEQKIGSVSHNASLLAAEVEGFTMLSTNGVKFDHSTESSLWAEI
PGSSKNAYHVHDQKFYVGQPRGAD-
VITHKLTYSRIDSDVPDSVATGDRLINDVDDQAQAAIPQRSIQEHDFKLVPNSQFHDHSGSQTAASIAQVDNKPKDG
GASTNELGELKKLDSFGRWMDKEIGGDCDDSLMASDSGNYWN-
TLGPENEDKEVSSLSHHMQLDIESLGPSLSQEQLFSIRDFSPDWAYSGVETKVLIVGTFLGSKKFSSETKWGCMFGEIEV
SAEVLTDNVIRCQAPLHATGQVPFYVTCRNRLACSEVREFEYRENASRVA-
SISNSSLQEEEQRFLVRLAKLLHLGLEKKWLNCSIERCSKCKIRSTLYSMRNNIDNELARAKESWMVSEVNFTDARDK
FIQSLLSDKLFEWLVCKVHGEGKGPDMLD-
GEGQGVIHLTAGLGYQWAMGLIVAASNNPNFRDAQGRTGLHWASYFGREETVIELVRLGVDPTLVDDPTSAFPGG
QTAADLASSQGHKGIAGFLAEAFLTSHLSSLNIKENITDTIDATIAAEKPTEAAAQVAFPLD-
GGADDGFSLKGTLAAVRKSTLAAALIQAAYRSSSFRYRQFPKSNDDSEVSLDLAALGPLNKYQRRSDFEDYLHSAAA
RIQQKYRGWKGRKEFLKIRNRIVKIQAHVRGRQVRRQYKKVIWSVSIVEKAILRWRR-
KRSGLRGFRLEKLCGDVIQGTEKTDEYEFLRIGRKQKFAGVEKALARVKSMVRDPVARDQYMRLVTKSENLKMNN
GEINVSPQDLS*
>MeCAMTA4.1 (DescChrB12G00091490.1)
MSQSGYDINVLFQEAQARWLK-
PAEVLYILQNHDKYQLTQEPPQKPTSGSLFLFNKRVLRFFRKDGHNWRKKKDGRTVGEAHERLKVGNVEALNCYY
AHGEQNSNFQRRSYWMLDPAYEHIVLVHYREIGEGKSTPGPAAQLSPGFSSSFSPSQTSYT-
TQNPDSTSAITDKYDPYRNSSSPSSIEVSSEMVTKENGLDATTEFTSYRKDEVSQYLRRLEEQLSLNEDSIKEIDPLCSEE
RATDNTELLEYEKQIPKEDNSANLLFRPEYFVNNQSY-
GGHAGMQLQTNNLVHLQDAGDSGKYDQSYLDKYADGNNESVSWNEVLDPSKASSGAEYQEKPQPSLRGPAEEHE
YSGWLNFNGTNARNSSLLLHQEVENFEIPAYAPVIGSHETNPDYYSM-
LYDPGQLGVPIEADSSLTVAQQQKFIIWEISPDWGFTSEATKVIVVGSFLCDPSQSAWTCMFGDTEVPTEIIQEGVLRC
QAPPHLPGKVTFCITSGNRESCSEVREFEYRAKSSCPHCSLSKTEVAK-
SPEELLLVRFVQFLLSDPSLQKEDSIETGIQLMRKLKTGDDSWGSIIEALLVGNGTSTGTVDWLLQQLLKDKLQQWLS
SKFQERQDQPSCTLSKKEQGIIHMVAGLGFEWALSPILSHGVSID-
FRDINGWTALHWAARFGREKMVAALIALGASAGAVTDPTSQDPIGKTPASIAANSGHKGLAGYLSEVALTSHLSSL
TIEESELSKGSAEVEAERTVDAISKGSFAVSEDQVSLKDTLAAVRNAAQAAARIQSAFRAHS-
FRKRQEREAATSAYSIDEYGVNSSDIQGLSAMSKLAFRNARDYNSAALSIQKKYRGWKGRKDFLAFRQKVVKIQAH
VRGYQVRKHYKVICWAVGILDKVVLRWRRKGAGLRGFRNEAEPND-
NDDESEDEDILKVFRKQKVDVAIGESVSRVLSMVDSPEARLQYHRMLERYRQAKAELGETSEAAATSLADMENDD
MYHFQ*
>MeCAMTA3 (DescChrB12G00106750.1)
MAEARSSPIGNQLDILQILAEARHRWLRPAEICEIL-
RNYKQFRVAPEPQHRPPSGSLLLFDRKALRYFRKDGHNWRKKKDGKTVKEAHEKLKSGSVDVLHCYYAHGEGNE

NFQRRSYWMLEEELSHIVLVHYRDVKGNRANFNNVKEHEDTIPCTQEIEDTVPHSEM-
DTSVSSNFHHDNYQVPAQTADTISMNSAQASEYEDAESVYNHQASSGLHLFLEEQQPAREKIDASLRDRHDPVSLSS
GYEGKLSAVPGRDFFSLAHADKAEDTEGASSTFQLQKHFDLPCWKDILENFTPGIE-
SASFQPQFSSQGDTAGIIPKQEDAIPKQLLSNSLPLQKEWQNLEDASSHLSKWAMDQKLHPNSTADLTTSFHEQELLS
GDLTNTLELFGTQKSGHSVQNDVQLQYSNTDQSITPEGKSIYSSTVKQLSEE-
GLKKLDSFSRWMSRELGDVNESHMQSSSGPYWDAVENENGIDDPKISSRVHLDTYLLGPSLSQDQLFSIIDFSPTWAY
VGSKIKVLIAGRFLKTPEEVENCKWSCMFGEVEVQAEVIADGVLRCQTPLNK-
AGMVPFYVTCSDRVACSEVREFEYRLSQDVNIIDSYSSSSSVMRFGKLLSLNSLSLPKCNTSNIVENTQLSNKISSLLKV
HNEEEWNKMLKLTSEAGVSLEKVKEEL-
LQKLLKDRLHVWLLQKAAEGGKGPSVLDEGGQGVLHLAAALGYDWALEPTIVAGVSVNFRDINGWTALHWAAS
CGRERTVASLVFLGAAPGALTDPTPKYPTARTPADLASANGHKGIAGYLAESAL-
SAHLFSLNLDKQDGDVAGVPEAKAGQIVPEHSTSVNSDGDLPYGLSLKDSLAAVYNATQAAARIHQVFRVQSFQK
KQLKEFGDDKYGMPHEHALSFIASKALKSGQHDEPVHAAATRIQNKFRSWKGRKE-
FLIIRQRIVKIQAHVRGHQVRKNYRKIVWSVGIVEKVILRWRRKGSGLRGFKSEALIGGPSMQDRSPKEDDYDFFKEG
RKQTEARSQIALARVKSMHQYPEARDQYRRLLNVVTEIQETKLVGDQINNSEATAEFD-
DLIDVEALFDDDTFMPAAS*
>MeCAMTA4.2 (DescChrB13G00136590.1)
MSQSGYDINVLFQEAQTRWLKPAEVLYILQNHEKYKFTHEP-
PHKPTSGSLFLFNKRVLRFFRRDGHNWRKKKDGRSVGEAHERLKVGNVEALNCYYAHGEQNPNFQRRSYWMLDP
EYEHIVLVHYREVGEGKSTPRSAVQLSPGLSSAFSPSTTSHTTHNRDSTSAVSDLYDPDRS-
SSSPSSTEISSEIVTKDNGLETLTGFTSSPKDGVSQFLRRLEEHLSLNEDSIKETDPLCSEEGITNDPELLEFAKQISEKDHY
VNMLHGPENIVNNQCYDFGEPPGLQLQSNNVVHLQDTGDGGKYHQPFVEYADG-
SKESISWNEVLESCKVSSGVDYQEKPQPSLREPAEEHEYSHWLNFNGNNVRNSSELLPQDVENFDIPLYSPVLGTHET
NPDYYSMLYDEGHLGVPIEPDSSLTVSRQQKFTIREISPEWGFTSEATKVIIVGS-
FLCDPSESAWKCMFGETEVPTEIIQEGVLCCVAPPHLPGKVTFCVTSGNRESCSEVREFEYRAKSSCPHCNLTQMEVA
KGPEELLLLVRFVQMLLSGSSMQKEDSIETGIQLLRKLKTDDGLWS-
RIIETLLIGNGTSTGTIDWLLEQLLKDKLQQWLSFKSQERRDQPSWTLSKKEQGIIHMVAGLGFEWALSPIISQGIGVN
FRDINGWTALHWAARFGREKMIAALLAFGASAGVVTDPTSQDPVGKTPASI-
AADSGHKGLAGYLSEVALTSHLSSLTLGESELSKGSAEVEAEKTVDSISKGSFSANEDQVSLKDTLAAVRNAAQAAA
RIQAAFRAHSFRKRQKEAAMLANSIDEYGLNSSDIHEVSAMSKLAFGNAHDYKSATLYIQK-
KYRGWKVRQDFLAFRQKVVKIQAHVRGYQVRKRYKVICWAVGILEKAVLRWRRKGVGLRGFRNEGEAIEDSEDED
ILKLFRKQKVDAAIEEAVSRVLSMVDCLEARQQYRRMLERYREAKAEVI-
ETSEAAATSADMENDDILPFQGGAASNYLT*
>MeCAMTA6 (DescChrB03G00618330.1)
MESGVQGLLVGSDIHGFHTLQDLDYGKIMEEASSRWLRPNEIHAILCNYKYFVIN-
VKPVHLPKSGVIKLFDRKKLRNFRKDGHNWKKKKDGKTVKEAHEHLKVGNEERIHVYYAHGEDNPTFVRRCYWL
LDKTLEHIVLVHYRETQEVQGSPVTPVNSNSSSVSE-
QSPWLLSEEFDSRAGHAYYVGVKEAIDPGDCLTVRNHEMRLHEINTLEWDELVTNDLNNSHMPKEDKNMSFDQH
NQIAVNGSRNDGIPLPVYNLSAEVPPLDYLTEPIARNNTSCLNIPEDAYSKAT-
KVQGNSDVQEKDARILGTGDSLDVLVNDGLQSQDSFGKWINDIIADSAGSVDNALVESSISSHDSHTSPAIDQLQSS
VPEQIFVITDISHTWAFSAEMTKILVTGYFHEQFLHLAKLDLCCVCGDACVRAEIVQAGVYR-
CLVPQHSPGLVDLFLSLDGHKPISQVLNFEYRPPLRDPVVSLEDKPKWDEFKLQMRLACLLFSTSRSLSILTSKVSPAN
LKEAKKFAHKTSSIFHSWTYLNKSIEDDRVSFSQAKDGFFELT-
LQNMLKEWLLERVVEGRKTTEYDAQGQGAIHLCALLGYTWAIHLFSWAGLSLDFRDKHGWTALHWAAYYGREK

MVAVLLSAGAKPNLVTDPTSEHPGGCTAADLASAKGYD-GLAAYLSEKSLVAQFKDMSLAGNVSGSLQTSSTDSINSANLSEEQLYLKDTLAAYRTAADAAARIQAAIKEHSLKVRTKAVQVANPEDEARNIVAAMKIQHAFRNYETRKRMAAAARIQHRFRTWKIRKEFLNMRR-VAIRIQAAFRGFQVRKQYRKIVWSVGVLEKAILRWRLKRKGFRGLHVDPIEVVADEREENDAEEDFFQVSRKQAEERVERAVVRVQAMFRSKKAQEEYRRMKLTCNQAELEYGGLLDHDIDLDR*

The GFF3 annotation file for the six *MeCAMTA* genes

ChrA06 maker gene 2317814 2333329 . + . ID=DescChrA06G00699830
ChrA06 maker mRNA 2317814 2333329 . + . ID=DescChrA06G00699830.1; Parent=DescChrA06G00699830
ChrA06 maker five_prime_UTR 2317814 2318041 . + . Parent=DescChrA06G00699830.1
ChrA06 maker CDS 2318042 2318081 . + 0 Parent=DescChrA06G00699830.1
ChrA06 maker CDS 2319627 2319746 . + 2 Parent=DescChrA06G00699830.1
ChrA06 maker CDS 2320884 2320999 . + 2 Parent=DescChrA06G00699830.1
ChrA06 maker CDS 2321973 2322061 . + 0 Parent=DescChrA06G00699830.1
ChrA06 maker CDS 2324676 2324718 . + 1 Parent=DescChrA06G00699830.1
ChrA06 maker CDS 2324852 2324978 . + 0 Parent=DescChrA06G00699830.1
ChrA06 maker CDS 2325470 2325595 . + 2 Parent=DescChrA06G00699830.1
ChrA06 maker CDS 2325692 2325993 . + 2 Parent=DescChrA06G00699830.1
ChrA06 maker CDS 2326766 2327296 . + 0 Parent=DescChrA06G00699830.1
ChrA06 maker CDS 2329654 2330361 . + 0 Parent=DescChrA06G00699830.1
ChrA06 maker CDS 2330820 2331389 . + 0 Parent=DescChrA06G00699830.1
ChrA06 maker CDS 2331872 2332219 . + 0 Parent=DescChrA06G00699830.1
ChrA06 maker CDS 2332870 2332977 . + 0 Parent=DescChrA06G00699830.1
ChrA06 maker three_prime_UTR 2332978 2333329 . + . Parent=DescChrA06G00699830.1
ChrB03 transdecoder gene 33021943 33029751 . - . ID=DescChrB03G00619160
ChrB03 transdecoder mRNA 33021943 33029751 . - . ID=DescChrB03G00619160.1; Parent=DescChrB03G00619160
ChrB03 transdecoder five_prime_UTR 33029564 33029751 . - . Parent=DescChrB03G00619160.1
ChrB03 transdecoder five_prime_UTR 33029382 33029433 . - . Parent=DescChrB03G00619160.1
ChrB03 transdecoder CDS 33029342 33029381 . - 0 Parent=DescChrB03G00619160.1
ChrB03 transdecoder CDS 33028721 33028840 . - 2 Parent=DescChrB03G00619160.1
ChrB03 transdecoder CDS 33028440 33028555 . - 2 Parent=DescChrB03G00619160.1
ChrB03 transdecoder CDS 33027599 33027687 . - 0 Parent=DescChrB03G00619160.1
ChrB03 transdecoder CDS 33027016 33027061 . - 1 Parent=DescChrB03G00619160.1
ChrB03 transdecoder CDS 33026392 33026671 . - 0 Parent=DescChrB03G00619160.1
ChrB03 transdecoder CDS 33025898 33026169 . - 2 Parent=DescChrB03G00619160.1
ChrB03 transdecoder CDS 33025127 33025510 . - 0 Parent=DescChrB03G00619160.1
ChrB03 transdecoder CDS 33024235 33024932 . - 0 Parent=DescChrB03G00619160.1
ChrB03 transdecoder CDS 33023344 33023920 . - 1 Parent=DescChrB03G00619160.1

ChrB03 transdecoder CDS 33022629 33022937 . - 0 Parent=DescChrB03G00619160.1
ChrB03 transdecoder CDS 33022217 33022261 . - 0 Parent=DescChrB03G00619160.1
ChrB03 transdecoder three_prime_UTR 33021943 33022216 . - . Parent=Desc-
ChrB03G00619160.1
ChrB12 maker gene 1187468 1195744 . + . ID=DescChrB12G00091490
ChrB12 maker mRNA 1187468 1195744 . + . ID=DescChrB12G00091490.1; Parent=Desc-
ChrB12G00091490
ChrB12 maker five_prime_UTR 1187468 1187837 . + . Parent=DescChrB12G00091490.1
ChrB12 maker CDS 1187838 1187850 . + 0 Parent=DescChrB12G00091490.1
ChrB12 maker CDS 1188033 1188158 . + 2 Parent=DescChrB12G00091490.1
ChrB12 maker CDS 1188262 1188377 . + 2 Parent=DescChrB12G00091490.1
ChrB12 maker CDS 1188998 1189086 . + 0 Parent=DescChrB12G00091490.1
ChrB12 maker CDS 1189361 1189406 . + 1 Parent=DescChrB12G00091490.1
ChrB12 maker CDS 1189824 1190316 . + 0 Parent=DescChrB12G00091490.1
ChrB12 maker CDS 1190954 1191072 . + 2 Parent=DescChrB12G00091490.1
ChrB12 maker CDS 1191300 1191387 . + 0 Parent=DescChrB12G00091490.1
ChrB12 maker CDS 1191483 1191706 . + 2 Parent=DescChrB12G00091490.1
ChrB12 maker CDS 1192485 1193161 . + 0 Parent=DescChrB12G00091490.1
ChrB12 maker CDS 1193768 1194350 . + 1 Parent=DescChrB12G00091490.1
ChrB12 maker CDS 1194554 1194856 . + 0 Parent=DescChrB12G00091490.1
ChrB12 maker CDS 1195169 1195249 . + 0 Parent=DescChrB12G00091490.1
ChrB12 maker three_prime_UTR 1195250 1195744 . + . Parent=Desc-
ChrB12G00091490.1
ChrB12 transdecoder gene 35877718 35886688 . - . ID=DescChrB12G00106750
ChrB12 transdecoder mRNA 35877718 35886688 . - . ID=DescChrB12G00106750.1; Par-
ent=DescChrB12G00106750
ChrB12 transdecoder five_prime_UTR 35886547 35886688 . - . Parent=Des-
cChrB12G00106750.1
ChrB12 transdecoder CDS 35886507 35886546 . - 0 Parent=DescChrB12G00106750.1
ChrB12 transdecoder CDS 35886239 35886358 . - 2 Parent=DescChrB12G00106750.1
ChrB12 transdecoder CDS 35886045 35886160 . - 2 Parent=DescChrB12G00106750.1
ChrB12 transdecoder CDS 35885763 35885851 . - 0 Parent=DescChrB12G00106750.1
ChrB12 transdecoder CDS 35884926 35884968 . - 1 Parent=DescChrB12G00106750.1
ChrB12 transdecoder CDS 35883107 35883314 . - 0 Parent=DescChrB12G00106750.1
ChrB12 transdecoder CDS 35882864 35882977 . - 2 Parent=DescChrB12G00106750.1
ChrB12 transdecoder CDS 35882467 35882759 . - 2 Parent=DescChrB12G00106750.1
ChrB12 transdecoder CDS 35881080 35881598 . - 0 Parent=DescChrB12G00106750.1
ChrB12 transdecoder CDS 35880248 35880933 . - 0 Parent=DescChrB12G00106750.1
ChrB12 transdecoder CDS 35879168 35879747 . - 1 Parent=DescChrB12G00106750.1
ChrB12 transdecoder CDS 35878705 35879028 . - 0 Parent=DescChrB12G00106750.1
ChrB12 transdecoder CDS 35877948 35878055 . - 0 Parent=DescChrB12G00106750.1
ChrB12 transdecoder three_prime_UTR 35877718 35877947 . - . Parent=Desc-
ChrB12G00106750.1
ChrB13 transdecoder gene 1480951 1488671 . + . ID=DescChrB13G00136590

ChrB13 transdecoder mRNA 1480951 1488671 . + . ID=DescChrB13G00136590.1; Parent=DescChrB13G00136590
ChrB13 transdecoder five_prime_UTR 1480951 1481327 . + . Parent=DescChrB13G00136590.1
ChrB13 transdecoder CDS 1481328 1481340 . + 0 Parent=DescChrB13G00136590.1
ChrB13 transdecoder CDS 1481449 1481574 . + 2 Parent=DescChrB13G00136590.1
ChrB13 transdecoder CDS 1481685 1481800 . + 2 Parent=DescChrB13G00136590.1
ChrB13 transdecoder CDS 1482467 1482555 . + 0 Parent=DescChrB13G00136590.1
ChrB13 transdecoder CDS 1482783 1482828 . + 1 Parent=DescChrB13G00136590.1
ChrB13 transdecoder CDS 1483187 1483685 . + 0 Parent=DescChrB13G00136590.1
ChrB13 transdecoder CDS 1484101 1484216 . + 2 Parent=DescChrB13G00136590.1
ChrB13 transdecoder CDS 1484479 1484566 . + 0 Parent=DescChrB13G00136590.1
ChrB13 transdecoder CDS 1484662 1484885 . + 2 Parent=DescChrB13G00136590.1
ChrB13 transdecoder CDS 1485646 1486325 . + 0 Parent=DescChrB13G00136590.1
ChrB13 transdecoder CDS 1487027 1487606 . + 1 Parent=DescChrB13G00136590.1
ChrB13 transdecoder CDS 1487826 1488119 . + 0 Parent=DescChrB13G00136590.1
ChrB13 transdecoder CDS 1488217 1488321 . + 0 Parent=DescChrB13G00136590.1
ChrB13 transdecoder three_prime_UTR 1488322 1488671 . + . Parent=DescChrB13G00136590.1
ChrB03 transdecoder gene 32254600 32264119 . + . ID=DescChrB03G00618330
ChrB03 transdecoder mRNA 32254600 32264119 . + . ID=DescChrB03G00618330.1; Parent=DescChrB03G00618330
ChrB03 transdecoder five_prime_UTR 32254600 32255095 . + . Parent=DescChrB03G00618330.1
ChrB03 transdecoder CDS 32255096 32255159 . + 0 Parent=DescChrB03G00618330.1
ChrB03 transdecoder CDS 32256312 32256437 . + 2 Parent=DescChrB03G00618330.1
ChrB03 transdecoder CDS 32257028 32257143 . + 2 Parent=DescChrB03G00618330.1
ChrB03 transdecoder CDS 32257696 32257784 . + 0 Parent=DescChrB03G00618330.1
ChrB03 transdecoder CDS 32258273 32258318 . + 1 Parent=DescChrB03G00618330.1
ChrB03 transdecoder CDS 32258786 32258918 . + 0 Parent=DescChrB03G00618330.1
ChrB03 transdecoder CDS 32259152 32259268 . + 2 Parent=DescChrB03G00618330.1
ChrB03 transdecoder CDS 32259384 32259442 . + 2 Parent=DescChrB03G00618330.1
ChrB03 transdecoder CDS 32260515 32260943 . + 0 Parent=DescChrB03G00618330.1
ChrB03 transdecoder CDS 32261689 32262383 . + 0 Parent=DescChrB03G00618330.1
ChrB03 transdecoder CDS 32262658 32263204 . + 1 Parent=DescChrB03G00618330.1
ChrB03 transdecoder CDS 32263520 32263828 . + 0 Parent=DescChrB03G00618330.1
ChrB03 transdecoder CDS 32263905 32263952 . + 0 Parent=DescChrB03G00618330.1
ChrB03 transdecoder three_prime_UTR 32263953 32264119 . + . Parent=DescChrB03G00618330.1
