# Supplementary figures and images for: Genome-Wide Identification and Expression Analysis of CAMTA Genes in Cassava Under Abiotic Stresses
Source: Plants (Basel). 2025 Dec 8;14(24):3743. doi: 10.3390/plants14243743 (PMC12736797; doi:10.3390/plants14243743)

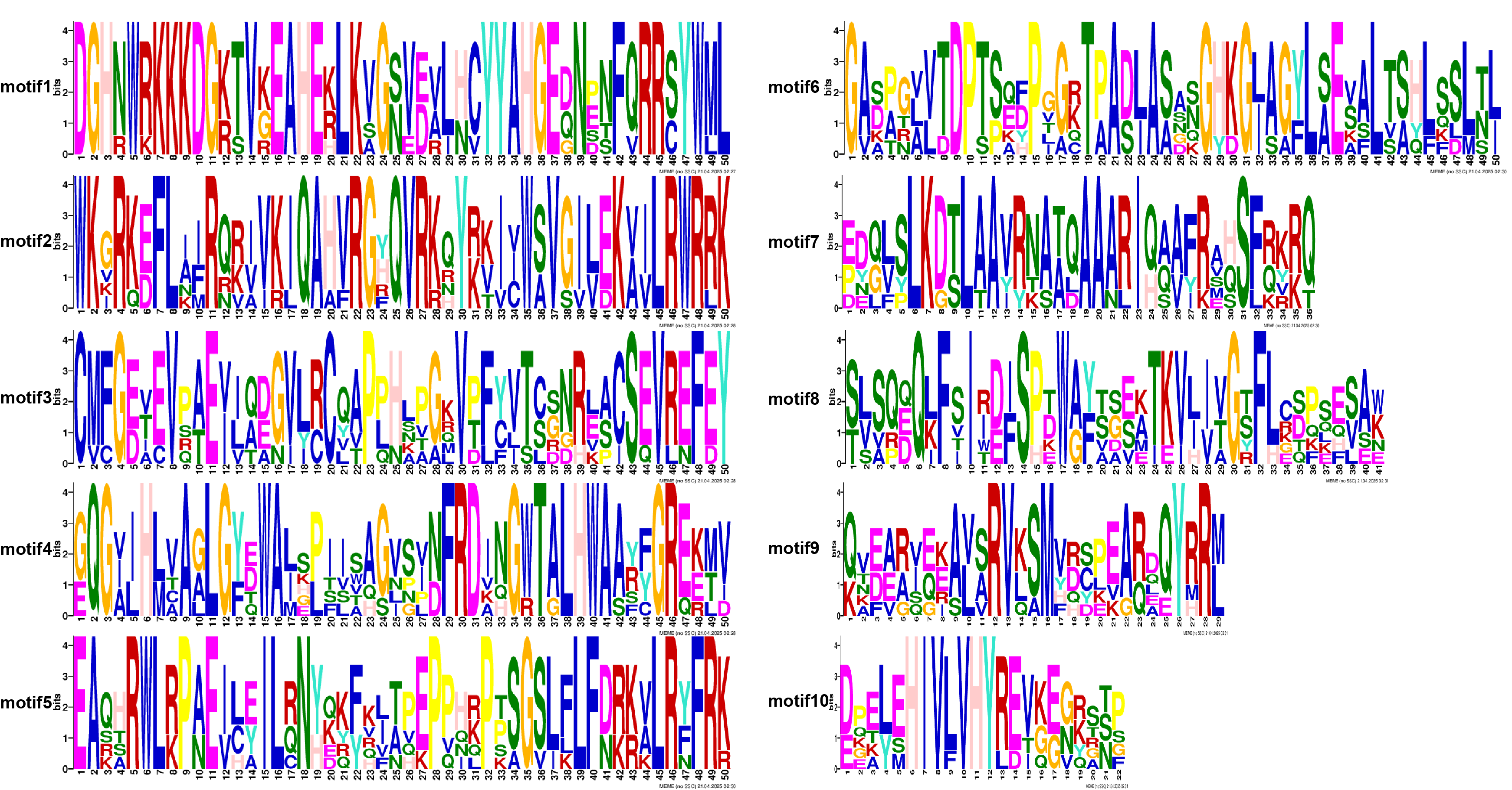

Supplement: Supplementary file 1 [file plants-14-03743-s001.zip › Figure S1.tif]

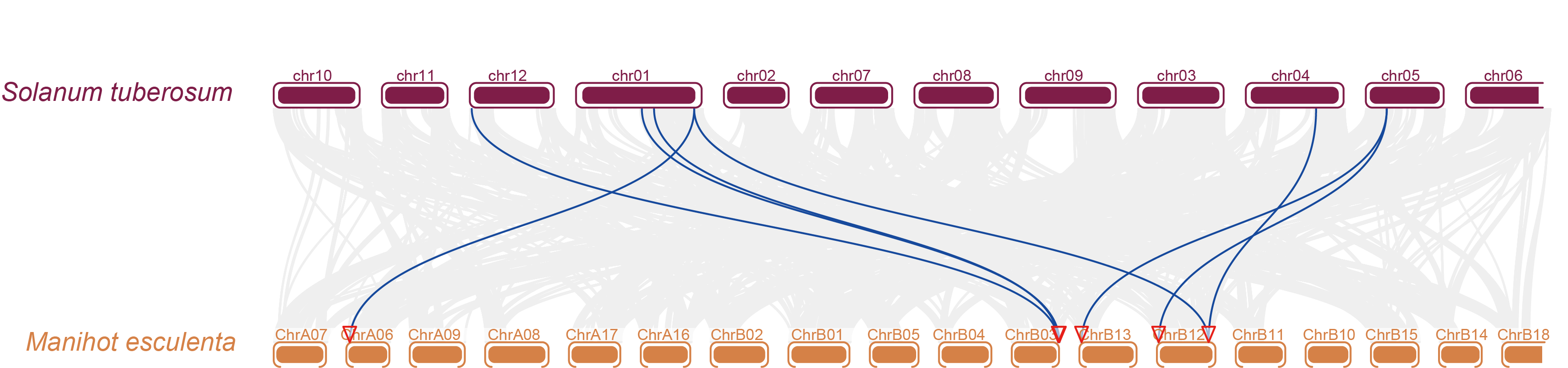

Supplement: Supplementary file 1 [file plants-14-03743-s001.zip › Figure S2.tif]

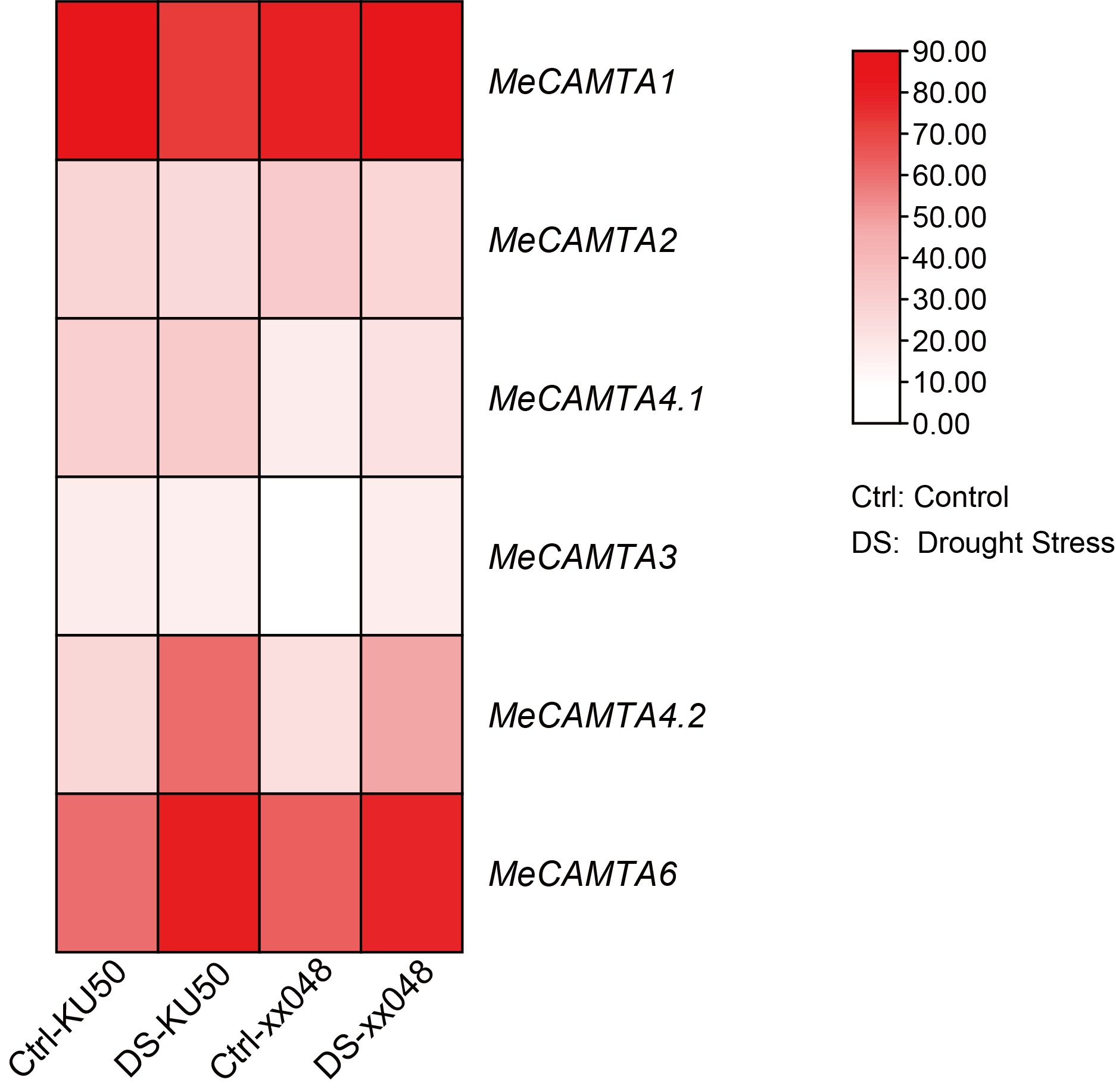

Supplement: Supplementary file 1 [file plants-14-03743-s001.zip › Figure S3.tif]
